# Supplementary material for: Antimicrobial resistance and population genomics of multidrug-resistant Escherichia coli in pig farms in mainland China
Source: Nat Commun. 2022 Mar 2;13:1116. doi: 10.1038/s41467-022-28750-6 (PMC8891348; doi:10.1038/s41467-022-28750-6)
Supplement: Supplementary file 1 — Supplementary Information [file 41467_2022_28750_MOESM1_ESM.pdf]

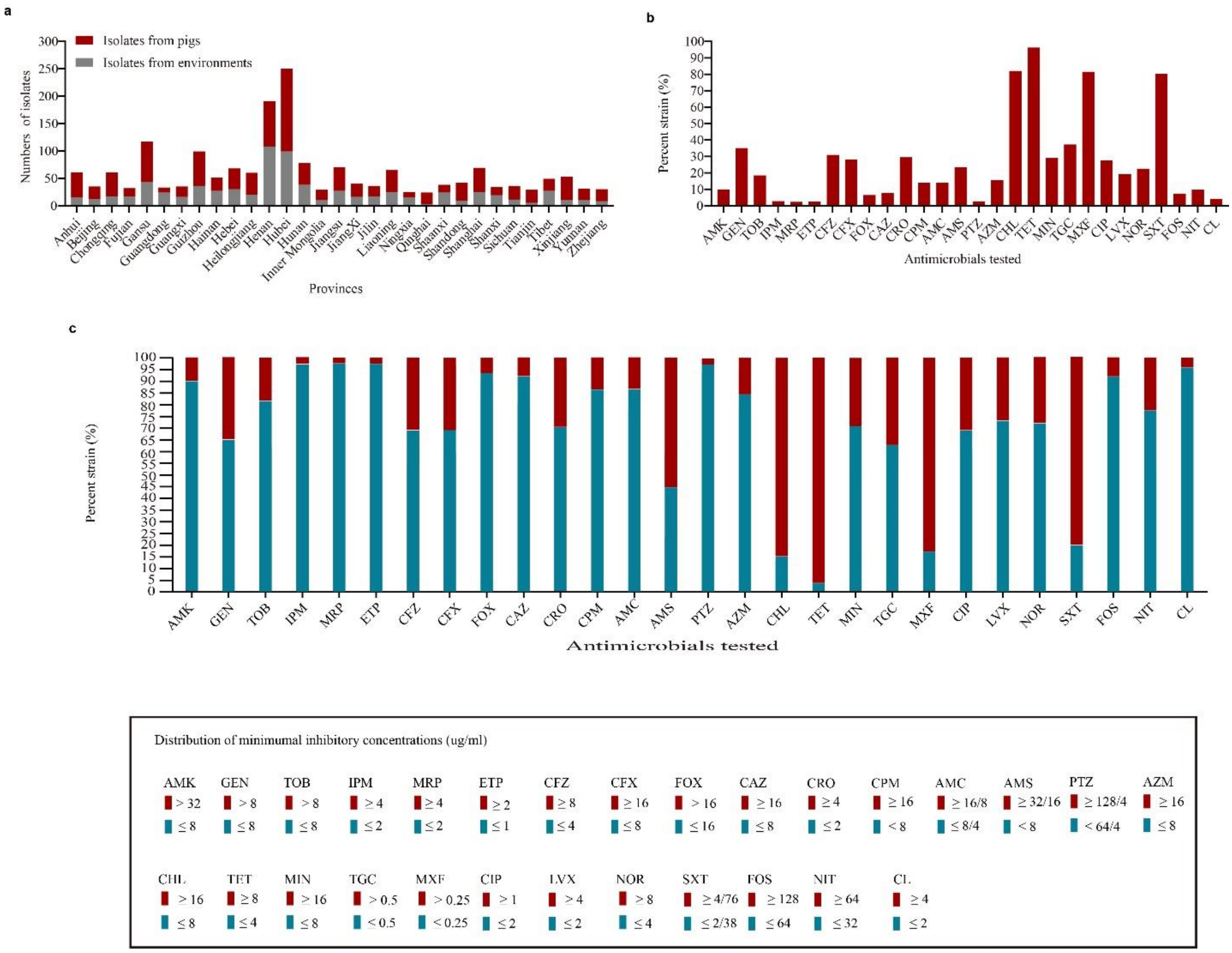

Fig. 1. **Isolation and antimicrobial resistance phenotypes of *Escherichia coli* isolates from pig farms in different provinces of China. a** Column chart showing the number of *E. coli* isolates from pig-associated samples and environmental samples collected from pig farms in each province in China; **b** Column chart showing the percentage of farm-originating *E. coli* isolates resistant to different antibiotics tested; **c** Column chart showing the distribution of the minimum inhibitory concentration (MIC) values of each of the antibiotics tested on *E. coli* isolates from pig farms in China. AMK: amikacin, GEN: gentamicin, TOB: tobramycin, IPM: imipenem, MRP: meropenem, ETP: ertapenem, CFZ: cefazolin, CFX: cefuroxime, FOX: cefoxitin, CAZ: ceftazidime, CRO: ceftriaxone, CPM: cefepime, AMC: amoxicillin/clavulanate, AMS: ampicillin/sulbactam, PTZ: piperacillin/tazobactam, AZM: aztreonam, CHL: chloramphenicol, TET: tetracycline, MIN: minocycline, TGC: tigecycline, MXF: moxifloxacin, CIP: ciprofloxacin, LVX: levofloxacin, NOR: norfloxacin, SXT: trimethoprim/sulfamethoxazole, FOS: fosfomycin, NIT: nitrofurantoin, CL: colistin.

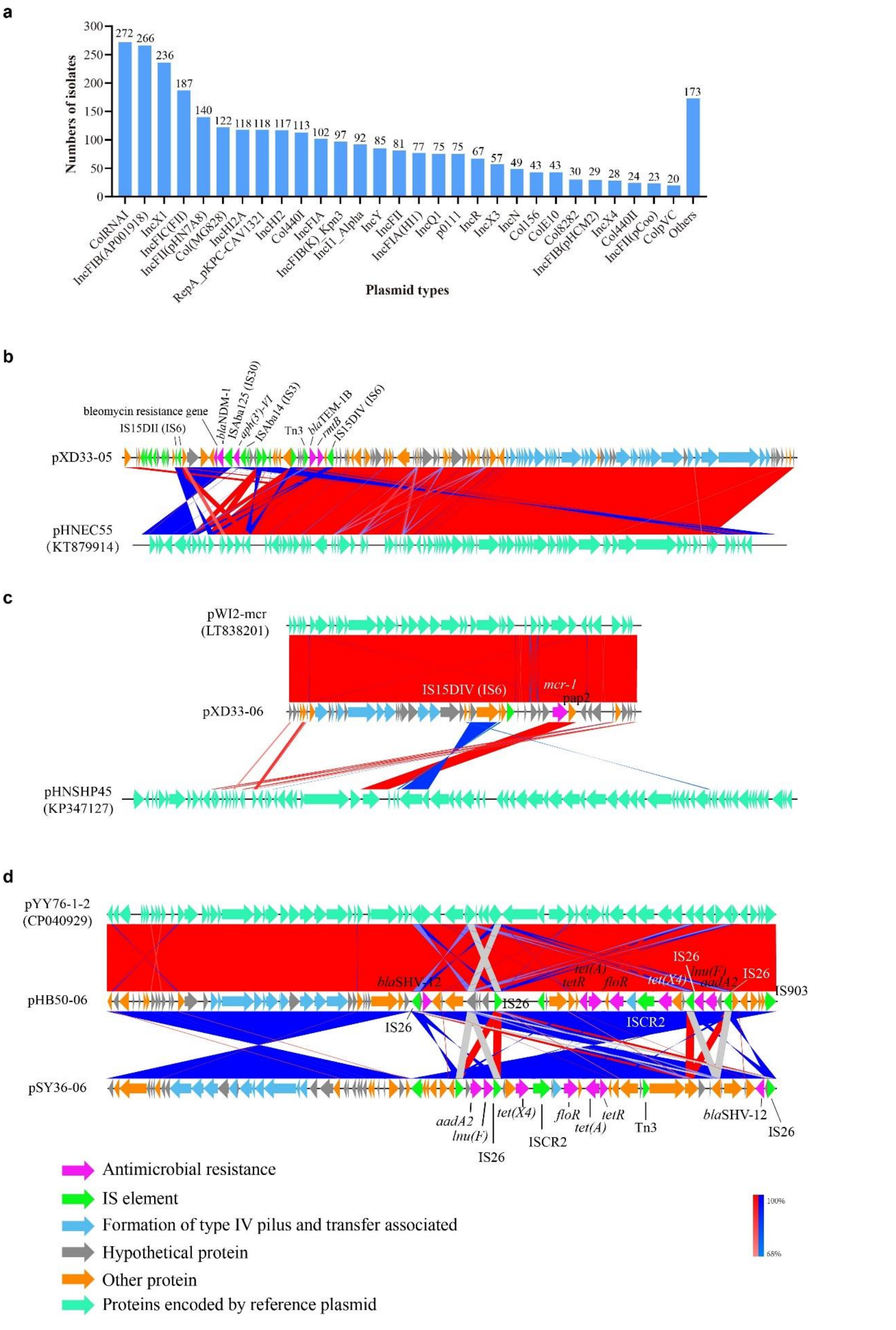

Fig. 2. **Plasmids mediating the dissemination of *bla*$_{\mathbf{NDM\text{-}1}}$, *mcr-1*, and *tet(X4)* identified in**

selected *Escherichia coli* isolates from pig farms in China. Panel **a** shows the distribution of different plasmid types identified in farm-originating extensively drug-resistant *E. coli* isolates in China; Panels B~D show the genetic structures of a bla_{NDM-1}-bearing plasmid (panel **b**), *mcr-1*-bearing plasmid (panel **c**), and *tet(X4)*-bearing plasmid (panel **d**). The color code represents the BLASTn identities of these regions between genomes. Arrows indicate putative coding sequences (CDSs) in different genomes.

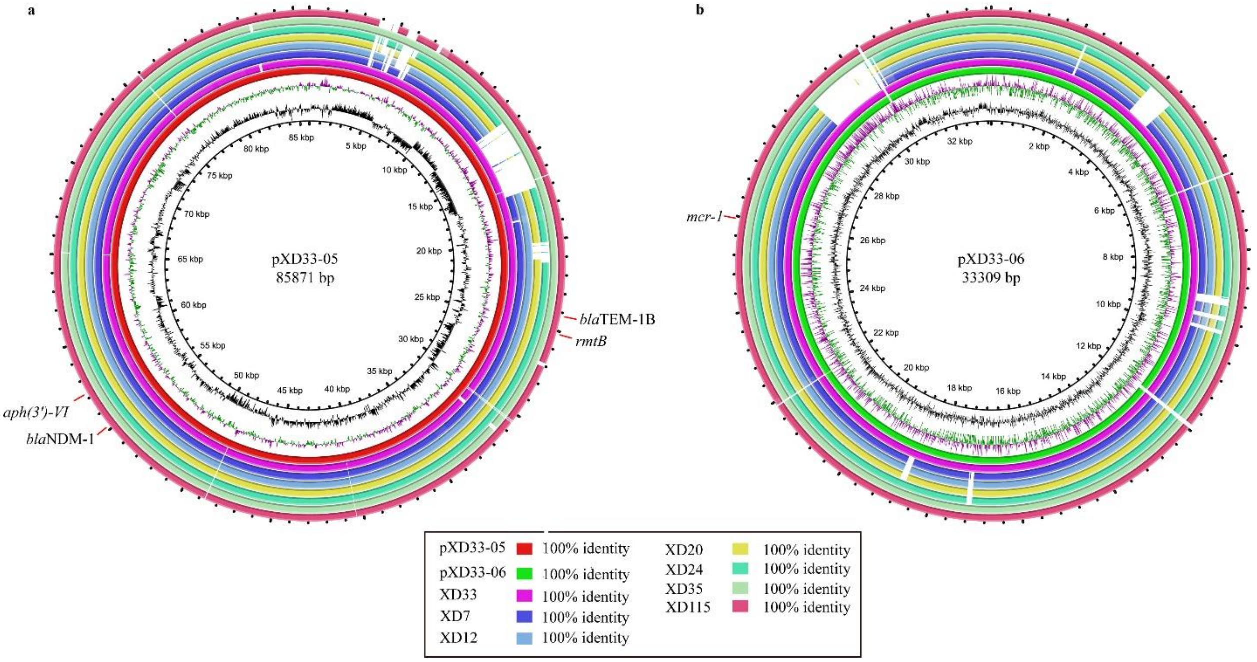

Fig. 3. **Comparison of the 85.9-kb IncFII-type-*bla*$_{\text{NDM-1}}$-carrying plasmid (pXD33-05; panel a) and the 33.3-kb IncX4-type-*mcr-1*-carrying plasmid (pXD33-06; panel b) with the genomes of New Delhi metallo-beta-lactamase (NDM) and mobilized colistin resistance (MCR)-coproducing *Escherichia coli* isolates from pig farms in China.**
