# Supplementary material for: Antimicrobial resistance and population genomics of multidrug-resistant Escherichia coli in pig farms in mainland China
Source: Nat Commun. 2022 Mar 2;13:1116. doi: 10.1038/s41467-022-28750-6 (PMC8891348; doi:10.1038/s41467-022-28750-6)
Supplement: Supplementary file 3 — Description of Additional Supplementary Files [file 41467_2022_28750_MOESM3_ESM.pdf]

Description of Additional Supplementary Files

File Name: Supplementary Data 1
Description: Sources and antimicrobial resistance phenotypes of 1871 *E. coli* isolates from pig farms.

File Name: Supplementary Data 2
Description: General features of sequenced multidrug-resistant *E. coli* isolates from pig farms in China.

File Name: Supplementary Data 3
Description: Virulence factor-encoding genes (VFGs) carried by sequenced multidrug-resistant *E. coli* isolates from pig farms in China.

File Name: Supplementary Data 4
Description: Antimicrobial resistance genes (ARGs) carried by sequenced multidrug-resistant *E. coli* isolates from pig farms in China.

File Name: Supplementary Data 5
Description: The class 1 integrase gene *intI1* carried by sequenced multidrug-resistant *E. coli* isolates from pig farms in China.

File Name: Supplementary Data 6
Description: Plasmid replicons identified in sequenced multidrug-resistant *E. coli* isolates from pig farms in China.

File Name: Supplementary Data 7
Description: Minimal inhibitory concentrations of different antibiotics when applied to transconjugants of carbapenem-resistant, colistin-resistant, and/or highly tigecycline-resistant *E. coli*.

File Name: Supplementary Data 8
Description: Numbers of single nucleotide polymorphisms (SNPs) identified between pig farm-originated multidrug-resistant *E. coli* isolates and human commensal *E. coli* collected across China.

File Name: Supplementary Data 9
Description: Sequence types of 287 human commensal E. coli collected across China analyzed in this study.

File Name: Supplementary Data 10
Description: The sizes of core genome alignments of each of the *E. coli* genome sequences compared to the complete genome sequence of *E. coli* XD35.

File Name: Supplementary Data 11
Description: Raw data for the MLST-based tree and its annotation codes used in iTOL (https://itol.embl.de/).

File Name: Supplementary Data 12
Description: Raw data for the Snippy tree and its annotation codes used in iTOL (https://itol.embl.de/).
