# Supplementary material for: Antimicrobial resistance and population genomics of multidrug-resistant Escherichia coli in pig farms in mainland China
Source: Nat Commun. 2022 Mar 2;13:1116. doi: 10.1038/s41467-022-28750-6 (PMC8891348; doi:10.1038/s41467-022-28750-6)
Supplement: Supplementary file 16 — Reporting Summary [file 41467_2022_28750_MOESM16_ESM.pdf]

nature research

Corresponding author(s): Xiangru Wang

Last updated by author(s): Jan 18, 2022

Reporting Summary

Nature Research wishes to improve the reproducibility of the work that we publish. This form provides structure for consistency and transparency in reporting. For further information on Nature Research policies, see our Editorial Policies and the Editorial Policy Checklist.

Statistics

For all statistical analyses, confirm that the following items are present in the figure legend, table legend, main text, or Methods section.

n/a	Confirmed	
☐	☒	The exact sample size (*n*) for each experimental group/condition, given as a discrete number and unit of measurement
☐	☒	A statement on whether measurements were taken from distinct samples or whether the same sample was measured repeatedly
☐	☒	The statistical test(s) used AND whether they are one- or two-sided<br>*Only common tests should be described solely by name; describe more complex techniques in the Methods section.*
☒	☐	A description of all covariates tested
☒	☐	A description of any assumptions or corrections, such as tests of normality and adjustment for multiple comparisons
☐	☒	A full description of the statistical parameters including central tendency (e.g. means) or other basic estimates (e.g. regression coefficient) AND variation (e.g. standard deviation) or associated estimates of uncertainty (e.g. confidence intervals)
☒	☐	For null hypothesis testing, the test statistic (e.g. *F*, *t*, *r*) with confidence intervals, effect sizes, degrees of freedom and *P* value noted<br>*Give P values as exact values whenever suitable.*
☒	☐	For Bayesian analysis, information on the choice of priors and Markov chain Monte Carlo settings
☒	☐	For hierarchical and complex designs, identification of the appropriate level for tests and full reporting of outcomes
☒	☐	Estimates of effect sizes (e.g. Cohen's *d*, Pearson's *r*), indicating how they were calculated

Our web collection on statistics for biologists contains articles on many of the points above.

Software and code

Policy information about availability of computer code

Data collection	Sequence reads from Illumina sequencing were de novo assembled via SPAdesv3.9.0; while short reads from Oxford Nanopore Sequencing were assembled and combined using the Unicycler v0.4.4 software with default parameters.
Data analysis	ResFinder 4.1 (https://cge.cbs.dtu.dk/services/ResFinder/) was used to determine putative acquired antimicrobial resistance genes (ARGs). PlasmidFinder 2.1 (https://cge.cbs.dtu.dk/services/PlasmidFinder/) was used to determine putative plasmids carried by the sequenced strains. SerotypeFinder 2.0 (https://cge.cbs.dtu.dk/services/SerotypeFinder/) was used to determined the in silico serogroups. Sequence types were identified with the program mlst in GitHub (https://github.com/tseemann/mlst) incorporating components of the PubMLST database (https://pubmlst.org/) . A minimal spanning tree was constructed using GrapeTree (https://www.grapetree.com/) version 1.5.0. A phylogenetic tree was also reconstructed based on the concatenated MLST alleles using fastMLST v0.0.15 (https://github.com/EnzoAndree/FastMLST), followed by multiple sequence alignment using MAFFT v7.407 (https://mafft.cbrc.jp/alignment/software/) and phylogenetic inference using FastTree (http://www.microbesonline.org/fasttree/). A phylogenetic tree was also generated based on the core genome SNPs using the snippy-multi program implemented in Snippy v 4.4.0 (https://github.com/tseemann/snippy). The MLST-based phylogenetic tree and the Snippy tree were visualized using the Interactive Tree Of Life (iTOL v.5). The RAST Sever was used for sequence annotation. The average nucleotide identities between two genome sequences were calculated by using the ANI calculator (http://enve-omics.ce.gatech.edu/ani/). A comparative genome analysis was performed and visualized using the BRIG package (http://brig.sourceforge.net/) and/or the EasyFig package (http://brig.sourceforge.net/).

For manuscripts utilizing custom algorithms or software that are central to the research but not yet described in published literature, software must be made available to editors and reviewers. We strongly encourage code deposition in a community repository (e.g. GitHub). See the Nature Research guidelines for submitting code & software for further information.

Data

Policy information about availability of data

All manuscripts must include a data availability statement. This statement should provide the following information, where applicable:

- Accession codes, unique identifiers, or web links for publicly available datasets
- A list of figures that have associated raw data
- A description of any restrictions on data availability

Whole genome sequences of E. coli isolates have been deposited into GenBank (BioProject accession no. PRJNA688628). GenBank accession numbers are given in Supplementary materials Table S1. Other data are available from the corresponding author upon reasonable request.

Field-specific reporting

Please select the one below that is the best fit for your research. If you are not sure, read the appropriate sections before making your selection.

☒ Life sciences ☐ Behavioural & social sciences ☐ Ecological, evolutionary & environmental sciences

For a reference copy of the document with all sections, see nature.com/documents/nr-reporting-summary-flat.pdf

Life sciences study design

All studies must disclose on these points even when the disclosure is negative.

Sample size

To objectively assess the antimicrobial resistance (AMR) profiles in pig farms in different provinces in mainland China, we randomly selected 2~3 different pig farms in each of the provinces to collect swabs from fresh feces and rectal swabs of pigs (with approximately 40 samples per farm), as well as swabs of drinking and fecal slurry, floors, troughs (for each point at least three samples per farm were collected). Through this strategy, a total of 2693 samples from pigs and breeding environments in 67 pig farms in all 31 provinces of mainland China were collected for E. coli isolation.

To understand the genomic characteristics of drug-resistant E. coli isolates from pig farms in different regions of China, we selected isolates (totally 515) with resistant phenotypes to either carbapenems (n = 49), colistin (n = 71), tigecycline (MIC value ≥ 4 μg/ml; n = 5), or broad-spectrum-cephalosporins (n = 495) for next-generation sequencing (NGS). These isolates also displayed resistance to aminoglycosides (n = 334), phenicols (n = 476), tetracyclines (n = 510), fluoroquinolones (n = 473), sulfonamides (n = 452), and/or nitrofurantoins (n = 59).

Data exclusions

No data were excluded from the analyses.

Replication

Antimicrobial Susceptibility Testing (AST) was performed following the Clinical & Laboratory Standards Institute (CLSI, United States) recommended microbroth dilution protocol (Performance Standards for Antimicrobial Susceptibility Testing, CLSI M100, 28th Edition). AST assays were repeated independently three times with similar results. All attempts at replication were successful.

Randomization

No randomization method was used. Because randomization is not relevant to this study.

Blinding

Data collection and Analysis was not performed blind. Because blinding has no effect on the experiment results.

Reporting for specific materials, systems and methods

We require information from authors about some types of materials, experimental systems and methods used in many studies. Here, indicate whether each material, system or method listed is relevant to your study. If you are not sure if a list item applies to your research, read the appropriate section before selecting a response.

Materials & experimental systems

n/a	Involved in the study	
☒	☐	Antibodies
☒	☐	Eukaryotic cell lines
☒	☐	Palaeontology and archaeology
☒	☐	Animals and other organisms
☒	☐	Human research participants
☒	☐	Clinical data
☒	☐	Dual use research of concern

Methods

n/a	Involved in the study	
☒	☐	ChIP-seq
☒	☐	Flow cytometry
☒	☐	MRI-based neuroimaging
